# Long Term Outcomes of Arthroscopic Shoulder Instability Surgery

**DOI:** 10.2174/1874325001711010133

**Published:** 2017-02-28

**Authors:** D. Karataglis, F. Agathangelidis

**Affiliations:** 1 Blue Cross Clinic, Thessaloniki, Greece; 2 First Orthopaedic Department, Aristotle University of Thessaloniki, Greece

**Keywords:** Bankart lesion, Bristow procedure, Latarjet procedure, Long-term outcomes, Shoulder arthroscopy, Shoulder instability

## Abstract

**Background:**

Anterior shoulder instability has been successfully managed arthroscopically over the past two decades with refined “anatomic” reconstruction procedures involving the use of anchors for the repositioning and re-tensioning of the antero-inferior capsuloligamentous complex, in an effort to recreate its “bumper effect”.

**Methods:**

Research and online content related to arthroscopic treatment of shoulder instability was reviewed and their results compared.

**Results:**

The short- and mid-term results of this technique have been very satisfactory. The greatest number of recent reports suggests that long-term results (>5 years follow-up) remain rather satisfactory, especially in the absence of significant glenoid bone loss (>20-25%). In these studies recurrent instability, in the form of either dislocation or subluxation, ranges from 5.1 to over 20%, clinical scores, more than 5 years after the index procedure, remain good or excellent in >80% of patient population as do patient satisfaction and return to previous level of activities.

As regards arthroscopic non-anatomic bony procedures (Latarjet or Bristow procedures) performed in revision cases or in the presence of >20-25% bone loss of the anteroinferior aspect of the glenoid, recent reports suggest that their long-term results are very satisfactory both in terms of re-dislocation rates and patient satisfaction.

**Conclusion:**

It appears that even “lege artis” performance of arthroscopic reconstruction decelerates but does not obliterate the degenerative procedure of dislocation arthropathy. The presence and grade of arthritic changes correlate with the number of dislocations sustained prior to the arthroscopic intervention, the number of anchors used and the age at initial dislocation and surgery. However, the clinical significance of radiologically evident dislocation arthropathy is debatable.

## INTRODUCTION

Anterior first time shoulder dislocation is almost always trauma-related. Despite prompt reduction and immobilisation for a reasonable but not excessive period of time before further rehabilitation, it can often lead to recurrent shoulder instability [1-3]. It appears that 55.7% of the overall population will develop shoulder instability within two years following the initial traumatic dislocation and this increase by a further 11% by the end of the fifth year [3]. Young males involved in sporting activities, especially overhead, and/or with increased functional expectations seem to have a far greater risk of recurrence of instability in the form of re-dislocation, recurrent subluxation or even apprehension. Furthermore, Habermayer *et al*. [4], Porcellini *et al*. [5] and Yiannakopoulos *et al*. [6] have suggested that labral, ligamentous and osseous pathology in shoulder instability are time- and recurrence dependent. Therefore, it appears to be the sub-group of young athletic males engaged in demanding activities that may profit more from rather early operative management of shoulder instability [7, 8].

A Bankart lesion or various Bankart-variants appear to be the “essential lesion” in most instability cases and is most usually amenable to “anatomic” procedures that aim to reconstruct and recreate the anatomy of the antero-inferior capsule-ligamentous complex. Over the past 10 years refined arthroscopic reconstruction techniques involving the use of suture anchors have been developed and offer very satisfactory short- and mid-term outcomes that are comparable to classic open techniques. On the other hand, “non-anatomic” procedures such as the Latarjet or Bristow coracoid transfer or other “bone-block” procedures are being used all the more often to address patients with a combination of osseous and ligamentous injuries. These procedures have recently been performed arthroscopically by a number of experienced surgeons, with a very promising outcome [9-12]. In this study we will review the long term clinical outcomes of arthroscopic procedures (>5-year mean patient follow-up time), both “anatomic” and “non-anatomic”, aiming to investigate if they remain satisfactory over the course of time.

### Long-term Clinical Outcome Following Arthroscopic Bankart Repair with Suture Anchors

Initial clinical studies on the long-term clinical outcome (>5-year mean patient follow-up time) following arthroscopic Bankart procedures describe the clinical outcome in patient series where techniques involving the use of tacks and trans-glenoid sutures were employed and report relatively high re-dislocation rates [13] and have been gradually abandoned over the last decade. They have been replaced with more refined “anatomic” reconstruction procedures involving the arthroscopic use of anchors for the repositioning and retensioning of the antero-inferior capsule-ligamentous complex, in an effort to recreate its “bumper effect” and to re-tension the antero-inferior capsule including the anterior bundle of the inferior glenohumeral ligament Figs. (**1** & **2**). Therefore, only studies reporting on the long-term outcome following arthroscopic stabilisation with the use of anchors will be analysed in this review. Both metal and bio-absorbable anchors have been used very successfully in the treatment of shoulder instability, neither one providing superior clinical outcomes. However, the visibility of the drill holes was significantly greater after using poly-L-lactic acid polymer implants [14].

Kim *et al*. [15] report on the clinical outcome of 32 patients (non-athletes) treated with an arthroscopic Bankart reconstruction using either metal or bio-absorbable anchors and compared it with another group where a trans-glenoid technique was used. The mean follow-up time for the anchor group was 6 years and the clinical scores improved significantly (Rowe score improved from 30 to 90 and Constant score from 68 to 95) and the reported recurrence rate was 6% (re-dislocation occurred 34 and 51 months post-operatively), with a further 3% of patients having a positive apprehension test but no re-dislocation

Rhee and colleagues [16] on the other hand reported on the long-term (6 year) outcome of a relatively small number (12) of high-demand collision athletes treated arthroscopically using metal anchors and compared it with the outcome following an open procedure. In the arthroscopic group, although clinical scores improved significantly, the percentage of patients returning to the same level of activities without any limitation or with moderate limitations was 81,7% but the recurrence rate (either dislocation or subluxation) was 25% (4 out of 12).

Unlike Rhee and colleagues [16] Larain and colleagues [17] reported very positive outcomes following arthroscopic treatment of both acute and recurrent instability in high demand contact athletes using suture anchors. They included 39 and 121 rugby players with acute and recurrent instability respectively in this report; the patients were followed for a mean of 5.9 years (3.9 to 8.9) and clinical outcome, as well as recurrence rates were recorded. They report good or excellent results in >90% of patients in both the acute instability and the recurrent instability group, and a recurrence rate of 5.1% and 8.3% respectively, all occurring after return to competitive rugby. They conclude that, in the absence of humeral head and/or glenoid deficiencies of >25%, a HAGL lesion, a significant capsular tissue attenuation or poor soft tissue quality, arthroscopic reconstruction with a suture anchor technique can offer an excellent clinical outcome, even in a very demanding athletic population.

Castagna and colleagues in 2010 [18] analysed the clinical and radiological long-term outcome (mean: 10.9 years) in a series of 31 patients. Their clinical outcome was very satisfactory with a total of 77.3% of good and excellent results according to the UCLA, SST and Rowe scores and an overall satisfaction rate of 84%. 83.9% of their patients had no loss of external rotation and almost all returned to sports, although only 71% returned to their previous level of sporting activity. They reported 5 cases of atraumatic and a further 2 cases of traumatic re-dislocation (overall rate of recurrence: 22.5%). Most re-dislocations occurred in overhead or contact athletes and a further point of interest is that three out of seven patients with a recurrence were still satisfied with the operative outcome and were able to resume their previous level of activity. This publication evidently represents a rather early experience of the authors with arthroscopic suture anchor reconstruction of anterior instability of the shoulder, where only two anchors were used in each case in the 4.30 and 3.00 o’clock position (for the right shoulder). Further, more recent clinical series have made clear that the use of a minimum of three anchors, with the lowest as close to the 5.30 o’clock position as possible, provides a better clinical outcome at least as far as recurrence rates are concerned [9].

Porcellini *et al*. in 2007 [5] reported on the long-term clinical outcome following arthroscopic treatment of acute as opposed to chronic bony-Bankart lesions. They included 65 patients that were followed for a mean of 5.6 years and report a significant improvement in clinical scores in both sub-groups, with a clear advantage of the acutely managed patients both in terms of functional recovery and range of movement as well as regarding return to sporting activities. They conclude that outcomes are maintained over a long follow-up period, especially if diagnosis and treatment are early.

Plath and colleagues [19] in a very recent publication, report on the clinical outcome as well as the rate of dislocation arthropathy in a rather large population of instability patients treated arthroscopically and followed for a minimum of 10 years (mean follow-up time: 13 years). They conclude that the overall clinical outcome was satisfactory, reporting a median Constant score of 94 and minimal external rotation deficit as compared to the contralateral shoulder. They report however a recurrence rate of 21%, (although only 14% proceeded to revision surgery) and a 28% rate of moderate or severe dislocation arthropathy. One has to point out however, that in a substantial number of shoulders (14%) arthroscopic tacks were used. It has been well documented that the above implants have been correlated with increased re-dislocation rates, but the authors do not clarify if the above mentioned sub-group of patients had an increased recurrence rate as compared to the overall population of their study.

Van der Linde *et al*. [20] report an alarmingly high instability rate of almost 35% in a rather large and homogenous patient series and a follow up of 8-10 years. This publication has however been critisised [13] for failing to differentiate between true re-dislocation, subluxation and apprehension in provocative positions. The authors report a clear inverse correlation between the number of suture anchors used and the possibility of recurrent instability, in which point one has to note that in 63,2% of their patients only 2 suture anchors were used, a factor recently recognised to increase post-operative re-dislocation rates [9]. It also is important to note that 41,6% of the post-operative instability episodes reported in this study (10/24) were recorded in <2 years, while the remaining 58,4% occurred 2-5 years (7/24) or even >5 years (7/24) post-operatively.

### Non-anatomic Arthroscopic or Open Techniques

Arthroscopic or open techniques involving bone block procedures or coracoid transfer techniques are used more and more often in both revision and primary instability surgery, especially in the presence of >20-25% bone loss of the anteroinferior aspect of the glenoid [21, 22]. Recent cadaveric studies suggest that defects as big as almost 30% of the size of the antero-inferior glenoid can be adequately restored after coracoid transfer in a Latarjet procedure [23].

Schmid *et al*. [11] have reported very encouraging mid-to-long term clinical results (mean 38 months) following a Latarjet procedure performed for revision of failed arthroscopic shoulder stabilisation. They recorded no further dislocations and only two subluxations in 49 patients, as well as a big improvement in post-operative clinical scores and patient satisfaction. Arthroscopic Bristow-Latarjet combined with an arthroscopic Bankart procedure has been used by Boileau and coworkers (2014) in 79 patients with anterior instability and >20% bone loss of the glenoid. They report 98% shoulder stability at 3 years and a very satisfactory clinical outcome as regards range of motion, patient satisfaction and return to competitive sports. They also report a very low percentage (9%) of dislocation arthropathy following this procedure. Mizuno *et al*. have also reported a low recurrence of instability (4 out of 68 shoulders- 5.9%) in their long term results study of open Latarjet procedure [24].

Dumont *et al*. [10] have reported the first long term (>5-year) results following arthroscopic Latarjet procedure as initially described by L. Lafosse. They published the results of 62 patients (64 shoulders) with a mean follow-up time of 76.4 months (minimum 5 years) with no recorded post-operative true re-dislocations and only one patient with recurrent subluxations (overall rate of post-operative instability: 1.59%). They also reported very satisfactory post-operative clinical scores (WOS: 90.6% ± 9.4%) and a very high overall patient satisfaction.

### Dislocation Arthropathy

Arthritic changes in the shoulder joint following shoulder dislocation were first described by Samilson *et al*. in 1983, who introduced the term “dislocation arthropathy” for this pathology and classified it radiologically [25]. The classification was later modified by Buscayret *et al*. [26] and consists of stage 1 with presence of osteophytes smaller than 3 mm, stage 2 with osteophytes 3 to 7 mm and minor irregularity of the glenohumeral joint, stage 3 with osteophytes larger than 7 mm combined with joint space narrowing and finally stage 4 with complete glenohumeral joint space loss. The entity of dislocation arthropathy has initially been described in shoulders treated conservatively and the prevalence of moderate/severe arthropathy has been shown to increase by roughly 1% per year. Surprisingly, this trend does not seem to be reversed by (open) procedures, although surgical management, if performed properly (without creating excessive tightness of the anterior structures, with a subsequent limitation of external rotation), appears to decelerate the degenerative procedure [27].

No such evidence was available concerning the long term effect of arthroscopic anatomic procedures on the prevalence and evolution of dislocation arthropathy until recently, when a number of publications have demonstrated that the long-term incidence of moderate and severe dislocation arthropathy is similar to that reported following open anatomic procedures [18, 19, 28]. Furthermore, it has been suggested that glenohumeral chondrolysis can occur following arthroscopic surgery. This has been mainly associated with post-arthroscopic infusion of the joint with local anaesthetic and secondarily with the extensive intra-operative use of radiofrequency for capsulorraphy [29]. The presence and grade of arthritic changes correlates with the number of dislocations sustained prior to the arthroscopic intervention and the age at initial dislocation and surgery [19]. Moreover, the number of anchors used, but not their type (metal, peek or bio-absorbable) was positively correlated with both the presence and severity of the degenerative changes recorded. However, patients suffering a post-operative recurrence of instability did not have a higher frequency or grade of arthritic changes [19].

**Fig. (3):**
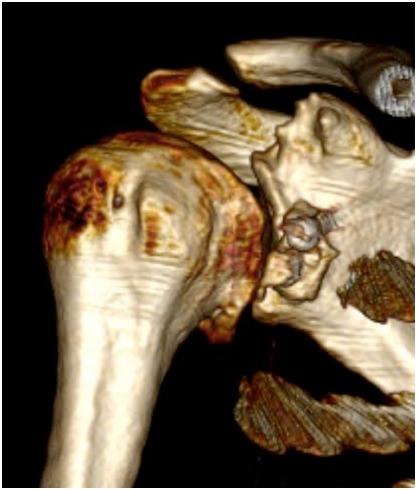
Stage 3 glenohumeral arthritis following arthroscopic Latarjet procedure.

Mizuno *et al*. reviewed the long term results of 68 shoulders treated with the Latarjet procedure. One fourth of the patients (23,5%) showed progression of arthritis [24]. This percentage is lower than other studies [27] and the authors assume that the subscapularis split approach may contribute to this compared with a subscapularis tenotomy during the procedure (Fig. **3**).

It appears that initial joint trauma sustained during anterior shoulder dislocation has long-term biologic effects on the shoulder joint cartilage and the joint physiology in general. Avoiding numerous pre-operative dislocations appears to prevent to some extent the occurrence and severity of post-operative dislocation arthropathy [19]. One should point out however that the clinical significance of radiologically evident dislocation arthropathy is debatable, as Constant score results do not seem to correlate with the presence or grade of postoperative dislocation arthropathy [8, 19].

## CONCLUSION

Anterior first time shoulder dislocation has significant short and long term effects on the shoulder joint. It can lead to anterior instability, loss of function and dislocation arthropathy. Open or arthroscopic anatomic procedures in the absence of severe glenoid bone loss provide good to excellent long term results even for the high demand athlete. Long term results of non-anatomic procedures in the presence of significant glenoid bone loss have been reported as satisfactory. Shoulders with a history of anterior dislocation are very likely to eventually develop some grade of arthritis even when they are treated surgically, however the presence of arthritis does not correlate with functional scores.

## Figures and Tables

**Fig. (1):**
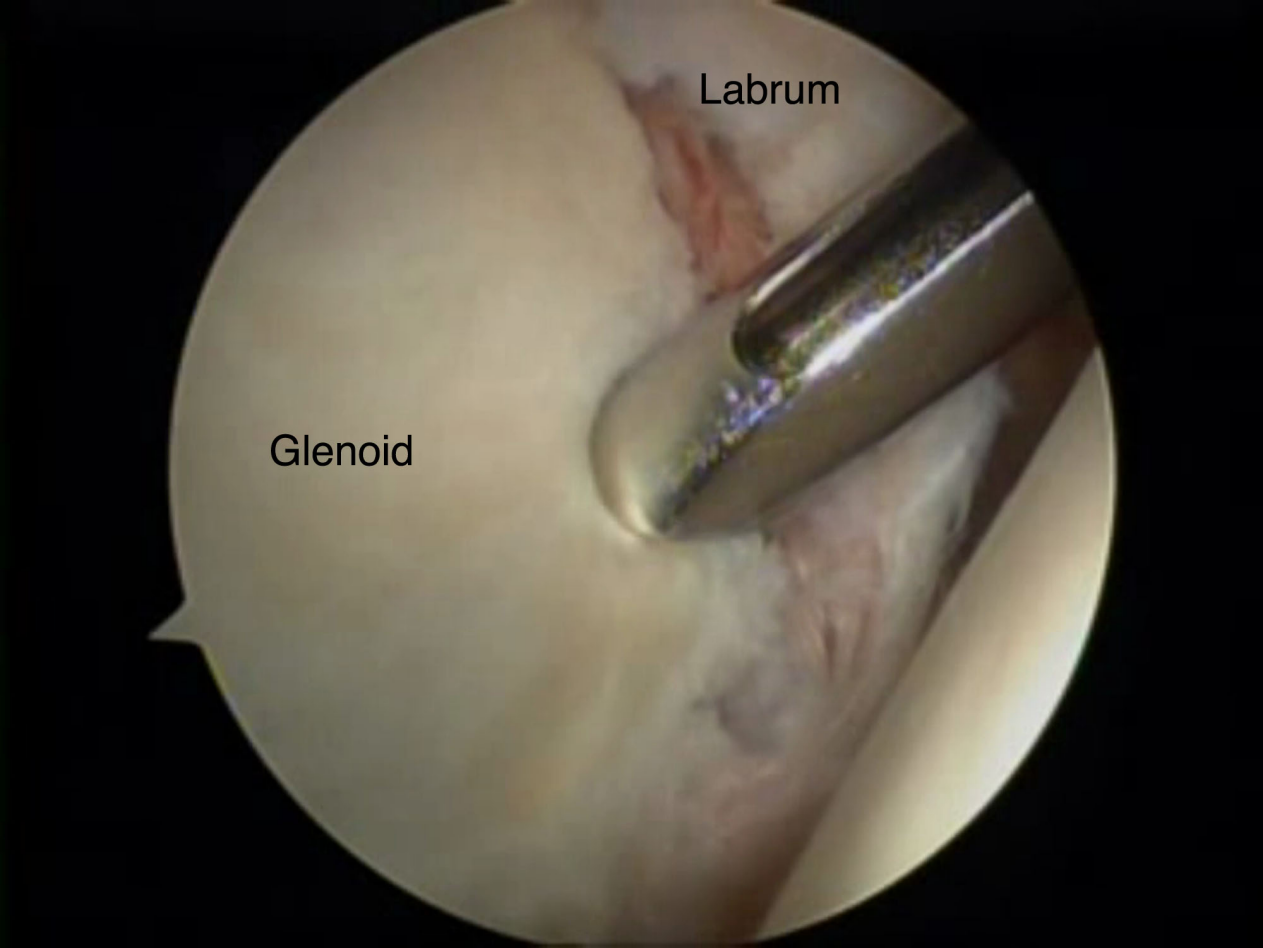
Bankart lesion, arthroscopic view from the posterior portal. The sheath used to introduce the suture anchor is located at the antero-inferior aspect of the glenoid.

**Fig. (2):**
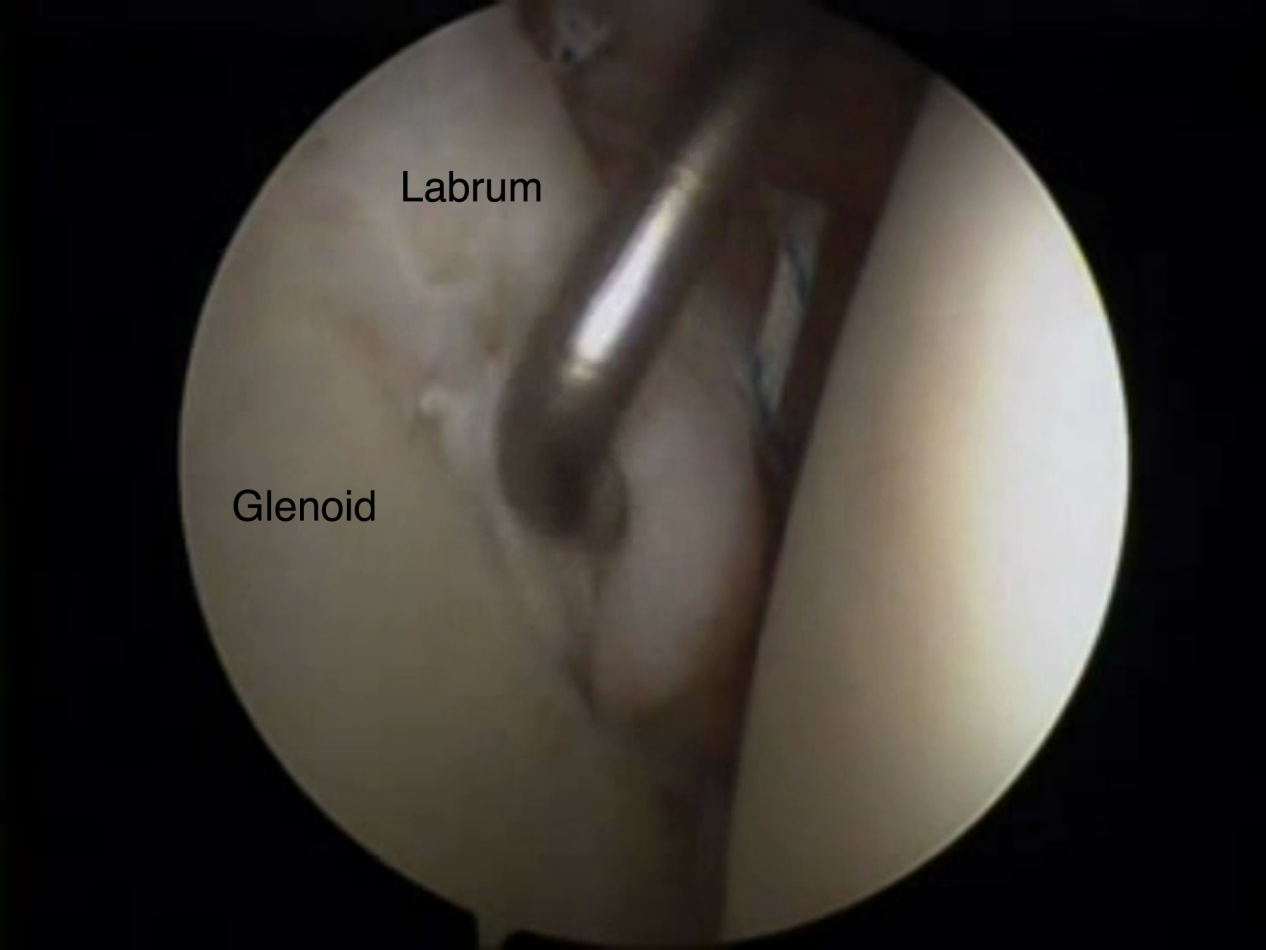
A Bankart lesion repaired arthroscopically with suture anchors. Arhroscopic view from the posterior portal. Probing of the labrum shows correct repositioning and re-tensioning of the antero-inferior capsuloligamentous complex, in an effort to recreate its “bumper effect”.
